# Experiences of Female Breast Cancer Survivors Concerning Their Return to Work in Spain

**DOI:** 10.3390/bs11100135

**Published:** 2021-10-02

**Authors:** Francisco Aguiar-Fernández, Yolanda Rodríguez-Castro, Mercedes Botija, Rosana Martínez-Román

**Affiliations:** 1 Faculty of Education and Social Work, University of Vigo, 32004 Ourense, Spain; francisco.aguiar@uvigo.es (F.A.-F.); rosana.mr@uvigo.es (R.M.-R.); 2 Faculty of Social Science, University of Valencia, 46003 Valencia, Spain; mercedes.botija@uv.es

**Keywords:** breast cancer, return to work, women, employment support

## Abstract

The objective of this study was to analyze the experiences of returning to work of women who had overcome breast cancer, identifying its physical and psychological consequences, the process they underwent, their motivations, and difficulties. A total of 19 female breast cancer survivors, with an age range of 30 to 57 years, participated in two focus groups. A semi-structured script was prepared about their experiences of returning to work. The results indicated that survivors’ self-perception was weakened by the physical and psychological consequences of the treatment of the disease; economic difficulties were one of the main reasons for going back to work; lastly, returning to work was a difficult process, mainly because of their physical/psychological limitations, the scarcity of job adaptation measures, and the limited support of the various public administrations. In addition, most of the women had to cope with seeking a new job without any guidance or job training. Significant difficulties related to the maintenance and return to work of female breast cancer survivors have been revealed. Findings highlighted the need to provide more and better information and guidance to cancer patients concerning their return to work or the search for a new job.

## 1. Introduction

Breast cancer is the most common type of cancer in women worldwide. In Spain, it is estimated that 32,953 new cases were diagnosed in 2020, and it constitutes 29% of all the new cancers diagnosed in the female population [1]. Survival five years after diagnosis has continued to increase in recent decades, as in all European countries, reaching 85.2% [2]. This greater chronification poses the challenge of examining not only the biological aspects, but also the psychological and social aspects throughout the process, especially when returning to everyday life, with employment being one of the most relevant aspects of this process.

Three groups of problems related to the psychosocial adjustment of women diagnosed with breast cancer have been identified [3]: (i) the emotional distress, which mainly leads to anxiety and depression problems, (ii) the sequelae that affect body image and self-esteem, and (iii) problems related to social adjustment, especially with regard to interpersonal relationships and other socioeconomic, occupational, or housing factors. Returning to work after cancer is a complex process that often involves changes or the loss of one’s job [4,5]. People who have survived cancer generally express a desire and a need to return to work, related to the restoration of identity and self-esteem [6,7,8], greater financial independence [9], the maintenance of social relationships [10], and feelings of recovery and the return to normalcy [11]. However, between 26 and 56% of cancer survivors lose their jobs or stop working during or after treatment [6] and they are estimated to have a risk of unemployment that is 1.4 times higher [12]. A meta-analysis of 26 international studies in the USA, Europe, and other countries showed that the unemployment rate is higher after breast cancer compared to other tumors [13].

### 1.1. Literature Review

Reviews of the international literature have identified a number of factors related to successful return to work in women with breast cancer, including: individual factors, factors related to the disease (prognosis, treatments, symptoms, and sequelae), or sociodemographic characteristics (age, education, income level); environmental factors (quality of support and services received), and; factors related to the workplace environment (the existence of job adaptation measures or the absence of discrimination) [11,14,15,16].

In Spain, the National Public Health System covers the necessary care and treatments for the entire general population at no cost. However, there are very few published studies on the impact of cancer on working life. Molina and Feliu’s [17] work reviewed the existing studies in Spanish samples focused on employment and on difficulties in cancer patients. They identified eight studies with a high variability in the prevalence of returning to work, between 16 and 70%. Although only three studies focused on females with breast cancer, they indicated that problems when going back to work after treatment were related to age, the location of the cancer, symptomatology, and the degree of support in the work setting [18,19,20]. A recent study concluded that around 40% of women diagnosed and treated for breast cancer reported loss in working capacity, and an important proportion of them attributed to the illness and its treatment that they had not returned to work, which is more associated to having a lower socioeconomic status [21].

In general, cancer is a process that involves a temporary disability (or medical sick leave). In Spain, in the case of breast cancer, the average duration is of 320 days, exceeding the 240 days of the estimated standard for combined tumors. When the legal deadlines for temporary disability run out, 47% of the patients maintain disability permanently, so only 53% of the women end up rejoining the working life [22]. However, the Spanish labor market is characterized by one of the highest unemployment rates in the European Union (16.13%) [23], and women are those who suffer the most unemployment (18.33% vs. 14.17% of men) and job precarity despite legislative progress in equality in recent years [24]. In 2020, 45.04% of women’s jobs were temporary, and of these, 59.99% on part-time [25].

### 1.2. Aim of This Study

Therefore, there is a large shortage of research on the job impact of cancer in the Spanish context and, to date, none has used a qualitative methodology to address the experiences of female survivors of breast cancer. The existing international literature limits the generalization of findings due to differences in health systems, labor laws, and social welfare systems in different countries. Thus, this study aimed to analyze the experiences of returning to work of women who had overcome breast cancer, identifying: the extent to which the physical and psychological consequences affected them; their personal motivations to return to work; the different options or pathways they chose to go back to work, and; the difficulties they encountered.

## 2. Methods

### 2.1. Ethical Approval

All subjects gave their informed consent for inclusion before they participated in the study. The study was conducted following the Declaration of Helsinki, and the protocol was approved by the Ethics Committee of the Ph.D. Program of Education and Behavioral Sciences (University of Vigo, Spain), (Project code: 011-2018). Furthermore, we have obtained the written informed consent of the participants for publication.

### 2.2. Participants

This study was qualitative. Its purpose was not to carry out a numerical measurement. Qualitative results allow obtaining the participants’ perspectives and points of view (their emotions, priorities, meanings, etc.). Its purpose was to “reconstruct” reality through the participants’ interpretations of their experiences [26]. Thus, in this study, the experiences of the participants allowed us to understand in greater depth the social behavior of breast cancer patients attempting to return to work. A total of 19 female cancer survivors, with age ranging from 30 to 55 years, participated in the study. The participants of this study belonged to a non-governmental association to support women diagnosed with cancer, located in northwestern Spain. All women had overcome breast cancer. Furthermore, all women had completed treatment.

All participants, before being diagnosed with cancer, were actively employed. Of the 19 women, 6 were civil servants, 2 were self-employed, 5 were household employees, and 6 were service sector employees. We separated the household employees and the service sector employees because they have different work regimes.

### 2.3. Data Collection

The first step was to contact the management of the non-governmental association to request its collaboration and explain the purpose of the study. The inclusion criteria to enter the study were: being a woman who was diagnosed with breast cancer and who had completed her treatment; it was also necessary for at least one year to have passed since her mastectomy operation and active treatment. In addition, before being diagnosed with cancer, the women were required to have been actively working.

The following procedure was used: the social worker of the association contacted the women who met the criteria of our study and asked them to participate voluntarily. We arranged a meeting with all of them in the association and explained the objective of the study. All those who attended the meeting signed the consent form to participate in the study. On another day, they were summoned to carry out the session. The method of data collection was the focus group (FG) because of its flexible nature, as it does not follow rigid scripts and tends to promote the exchange of opinions and experiences concerning the topic of analysis [27]. In addition, as Barbour [28] points out, focus groups have been very useful to deal with some “sensitive” topics, especially in health research. Concerning the formation of the groups, we tried to make them heterogeneous in terms of the age and socio-labor situation of the previously selected female participants. Following the recommendations of Barbour [29], with the 19 female participants, we organized two medium-sized focus groups of up to 10 people, as this was an adequate number for the participants to talk about the issues raised in a relaxed and informal environment, under the leadership of one of the study researchers. Thus, in this study, we had FG1, made up of 10 women, and FG2, which included 9 women.

The focus groups were carried out in the Association itself. For this study, all participants were asked their age and employment status before cancer, and an ad hoc script of semi-structured questions was prepared: How has your life changed since you overcame your cancer? What are the changes at the work level? Did you feel supported by your co-workers? Do you think jobs should be adapted? What measures should be taken at the work setting?

The two focus groups, with a maximum duration of 50 min, were recorded to conduct the subsequent transcription and analysis of the collected data. The focus groups were conducted out in Spanish and later translated into English. The coding used for the identification of each female participant in the focus group, to maintain anonymity, was: focus group 1 (FG 1) and the number assigned to each female participant (P1 to P10), and: focus group 2 (FG 2) and the number assigned to each female participant (P1 to P9).

### 2.4. Data Analysis

The ATLAS.ti. v.8 (Scientific Software Development GmbH, Berlin, Germany) program was used to organize, store, compare, and analyze the data obtained in the interviews in the different focus groups. The use of this software allows the simultaneous work of researchers [30], so that the data processing as well as its coding were triangulated. The triangulation of researchers of the analysis of the phenomenon provided greater strength to the findings. This reduces the biases of using a single researcher in data analysis and makes the findings more consistent [31]. The procedure followed was that each researcher independently carried out the analysis and its categorization, and, subsequently, these analyses were compared. Hence, the results reported in this study were the product of the consensus of the study researchers. The naturalistic content analysis was based on the words and expressions of the female survivors of cancer following five basic rules [32,33]: (i) Completeness: categorizing the entire content of the focus groups; (ii) Exclusivity: focusing on the same idea in the same category, without mixing different aspects; (iii) Semi-induction: carrying out a careful pre-categorization or coding of the phrases or expressions used by the survivors; (iv) Belonging to the category: assessing whether it was appropriate to maintain that category; and; (v) Objectivity: to comply with this rule, we crossed the encodings and categorizations of the content analysis of the focus groups among the members of the research team. Thus, through the content analysis of the focus groups, three main categories (themes) were created (see Table 1): (i) Self-perception of cancer survivors; (ii) Motivations for going back to work; (iii) Job reincorporation: the pathways. These main categories or themes were divided into secondary categories according to their level of depth, and will be developed in the results section.

## 3. Results

### 3.1. Theme 1: Self-Perception of Cancer Survivors

In this first category, we collected the personal image of each of the cancer survivors and their relationship with the surrounding world, as well as the impact that the disease had on their body and mind.

All participants openly expressed that physical changes in their bodies due to the treatments followed during the disease and after the operation impacted them severely, mostly at the psychological level. The fact that they gained weight, the removal of the breast, as well as the loss of hair, caused them to feel insecure and afraid of being rejected. Almost all the participants claimed that, due to the social pressure of their environment, they were forced to use a wig or prosthesis to disguise their illness. However, another of the sequelae they indicated were changes in their bodies after the treatment and operation. Lymphedema may become one of the most feared sequela of breast cancer treatment, as the appearance of the edematized arm may be more worrying than even the mastectomy itself. The absence of a breast can be disguised with prosthetic bras, whereas a disfigured arm is a constant memory of the breast cancer and sometimes arouses the curiosity of the social environment, especially the work setting, causing the women to feel insecure.

“You don’t feel valued if you don’t have a breast, your arm is disfigured, you feel rejected, it affects all of us in general, when you go out, and you feel different” (FG1, P1).
“I cried about my hair. It’s like seeing a ghost in the mirror” (FG1, P1).
“When people see you wearing a wig, they already label you as sick, they stare at you like you’re a freak. I felt terrible, horrible” (FG2, P6).
“The wig, in my case, gave me security” (FG2, P4).
“You have to accept that you’re no longer the same” (FG1, P1).

Generally speaking, in addition to the physical change in their body, cancer survivors claimed that they had numerous physical consequences. In relation to physical sequelae, they pointed out that they could not pick up weights, they tired more easily, they felt their legs swelling, and they could not raise their arms as they used to, especially those with lymphedema disorder in non-severe degrees.

“You are also weaker” (FG1, P1).
“But of course you don’t have the strength you used to” (FG2, P2).
“I get very tired, anything I do makes me tired” (FG1, P1).
“You never get back physically to how you were before. I cannot do what I used to do” (FG2, P6).
“Your arm swells up, they measured me and told me that it wasn’t important until the inflammation reached two centimeters. You try to ignore it and lead a normal life. But I’ve had some frights” (FG1, P4).

### 3.2. Theme 2: Motivations to Go Back to Work

This second main category included the arguments of breast cancer survivors regarding their motivations to go back to work. The motivations referred to in the two focus groups revolved around the positive view that allowed them to be able to go back to work, and economic necessity. On the one hand, the fact that they were discharged by the doctor so that they could return to work was synonymous with “overcoming” the disease. Cancer survivors saw the return to work as a return to normality in their lives before the disease. However, on the other hand, for more than half of the participants, their main motivation was the economic need. During their illness, operation, and radiation and/or chemotherapy treatments, their family economy was affected due to the increase in expenses of up to an average of EUR 400 per month, generated by the need to buy special bras, cotton clothing, a wig, special moisturizers for “radiated” skin, and to improve their eating habits, among others. Moreover, as they stated, access to benefits and financial aid of the public administration was difficult and insufficient, due to the requested requirements, scarce coverage, and excess red tape that often extended over time. This situation of economic precariousness had, in some cases, led to the impoverishment of the family nucleus, even having to resort to non-governmental institutions that deliver monthly batches of non-perishable food to families without resources.

“The doctors discharge you and tell you that you can work now. And you are very happy because you think that you have already overcome your illness. Going back to work means that you are cured” (FG1, P9).
“Economically, everything changes a lot, it reduces your economic level. In addition, practically nothing is paid for by Social Security; you also try to change your food. You go to a corsetry to order a special bra and it’s twice or three times as expensive as a normal bra. The moisturizer that was prescribed for me cost 20 euros a jar and it ran out very soon. Well, if you buy a wig, that increases the expenses by 1.000 euros. Only the prosthesis is paid for [by Social Security]” (FG1, P1).
“I go to the church for food, I’m not ashamed to say it but, of course, what they give you is insufficient, and the creams that they tell you to use where do you get the money for everything? I don’t think that 400 euros was enough to pay for the expenses” (FG2, P2).
“They help with the trips, but it is very slow and involves a lot of paperwork. And the aids do not arrive” (FG2, P2).

### 3.3. Theme 3: Going Back to Work: The Pathways and Difficulties

In this third main category, the participating women’s process of returning to work was discussed in the two focus groups. After the female cancer survivors were discharged and could go back to work, they found different pathways to follow, depending on the type of work. The six women who were permanently employed as civil servants had the opportunity to go back to their previous jobs, as well as the two women who were self-employed and working in a family business. However, the rest of the women, both those who worked in the services sector (six) and the domestic workers (five), had been dismissed during the period of recovery from their illness and had to cope with searching for a new job. These different situations show that returning to work depended to a large degree on the type of work they had. Those who had a permanent contract as civil servants and those who were self-employed went back to their jobs without any problem, whereas those who had temporary jobs were either fired or their contracts were not renewed.

“I tell you, I’m a civil servant, I had no trouble going back to work” (FG1, P1, l.86).
“Returning to work depends a lot on the type of job you have. If you have a job by contract, they do not renew it, as in my case” (FG2, P2).
“I used to work in a major fish company, but I couldn’t do that job now because my body would not be capable of working every night like before. My contract ran out during my recovery, and after six months of sick leave, they did not renew me” (FG2, P7).
“I lost my job and lost a number of offers because of the disease I used to have” (FG2, P8).

#### 3.3.1. Going Back to the “Same Job”

In the focus groups, the women alluded to the fact that, in order to return to their old job, they considered it essential to adapt the job. As mentioned above, they claimed they had physical and psychological consequences. Many women stated that they did not think they would be able to return to their previous job because they did not feel capable of it and they could not leave the responsibility of their work to their co-workers. Of the few women who had the option of returning, none of the jobs were adapted to their new needs.

“On the labor issue, I feel very atrophied. The doctors tell you that you cannot do anything, even if you are medically discharged. I even considered quitting work, even though I have a steady job” (FG1, P1).
“They don’t adapt anything to me. When I was discharged and the doctors said I could work, my colleagues are not going to do my job and they don’t need you just to sit in the office” (FG2, P9).
“To go back to the previous job, you need to job to be adapted” (FG2, P4).

#### 3.3.2. Searching for a New Job

After overcoming their illness, 11 of the female cancer survivors were seeking a new job, as they had been fired or their contracts had not been renewed during the period of sick leave. The difficulties these women expressed about going back to work, besides their physical difficulties, were overcoming the social stigma associated with cancer, as it created a great deal of mistrust in the employers. This situation caused several women to pose the dilemma of hiding the disease they had overcome in the job interviews.

“A friend of mine had cancer and when she started looking for work, she was already almost bald, so she went to an interview wearing a scarf (her hair never grew back). They asked her if she was bald, and she said yes; then, they told her that she was not suitable for that job. And that job was in a factory, it wasn’t working in a boutique facing the public. (FG1, P1).
“When I went to ask for a job in a bakery, they asked me if I was still sick because they were afraid I would ask for sick leave” (FG2, P4).
“The last time they told me, ‘O.K. that is alright, but how many check-ups are you going to get, are you going to get sick leave, you’re not going to take any sick leave”? (FG2, P6).
“Well, cancer is a disease like any other, it’s no shame. But at the employment level, you cannot tell them; otherwise, they do not hire you” (FG1, P10).

Another of the difficulties to which these 11 survivors frequently referred was the lack of work orientation, as well as the need to re-train in order to have access to a new job that was better adapted to their physical conditions. Of course, they also alluded to the lack of coordination between medical, psychological, and social services, because, from the medical point of view, they were discharged even though they were not psychologically prepared to face going back to work. Moreover, they thought that the fact that they were unable to carry out their previous job was not taken into account, and it would therefore be necessary to prepare them to search for a new job adapted to their new physical conditions.

“The Services’ enormous lack of coordination, by the (Health) Administration, you have to drag out the words from them, Social Security, Finance, Social Services” (FG1, P10).
“I think, there should be a place where there were workshops to learn how to defend ourselves in our life after the disease. For example, when looking for a new job, you should have some indications, some tools” (FG2, P2).
“Courses should be taught where we can do and learn things that are within our reach” (FG2, P8).

#### 3.3.3. Early Retirement

Another situation that had been detected was that women aged near 55 were pre-retired, although it is important to note that this was only true for women who had a permanent job as a civil servant. Thus, of the six women who were civil servants, two of them who were nearly 55 were granted early retirement, even losing their money. The rest of the women, aged near 55, who belonged to the service sector or were domestic employees were unemployed after overcoming the disease and were not given the opportunity to retire early, even though they did not feel strong enough to go back to work.

“I retired before I reached the right age, and was almost one year on sick leave” (FG2, P6).
“But they’re not going to give me anything. I have to go back to work” (FG2, P4).

## 4. Discussion

The results of this study indicated the significant difficulties related to job maintenance and return to work of female breast cancer survivors. These difficulties could occur at two different times: on the one hand, during the treatment itself, when most of the women were on sick leave due to disability or were receiving some form of social benefit, and; on the other hand, once this period was over, when they decided to go back to work or had to find a new job.

First, women recognized the existence of limitations related to their own self-concept and self-esteem, and to the physical (such as fatigue, reduced physical strength, pain, swelling, lymphedema, etc.) and psychological (anxiety, fears, etc.) consequences that impacted their ability to feel confident when they attempted to resume the different facets of everyday life, and that also limited their capacities for going back to work. This occurred especially in women with greater physical or psychological consequences and in jobs that required greater physical effort, in line with other international studies [15,33,34]. In the Spanish setting, it has also been found that anxiety and depression are especially significant at the time when women with cancer finish the treatments and resume their daily life [22,35].

Concerning the personal motivations to return to work, we detected that our participants considered it as a clear sign that they had recovered and that they had overcome their illness, so they could usually return to normal life, in line with what was pointed out in other studies [5,36]. In many of our women, however, we found that the main reason for returning to work was economic need. Our participants pointed out that, during their convalescence, their income had decreased, while there was an increase in expenditure of about EUR 300–400 per month. Despite the extensive Spanish health coverage, there are complementary treatments, of rehabilitation or prosthetics, which are not covered or only partly covered by Social Security. These data coincide with the recent report of the Spanish Cancer Association which notes that an average family can increase health expenditures by between 4 and 15%, with the average monthly expenditure being between EUR 150 and EUR 300 (the latter the figure corresponds to the average for breast cancer) [37]. This same study described as particularly vulnerable the cases of persons who receive unemployment benefits and subsidies, and self-employed people when the amounts do not reach the minimum inter-professional salary. In 2018, women accounted for 60.18% of new cases of cancer with incomes equal to or less than the minimum inter-professional salary (EUR 858.55 in 2018). Some women in our study also told of the difficult situations they underwent when they had to apply for non-contributory benefits or social services. Access was complicated due to the existence of rigid requirements and lengthy processes of paper work, and the amounts of the benefits were in many cases insufficient. Other international studies have also highlighted the impact that cancer and its treatments have on finances and the pressures survivors are under to return to work as a way of being able to support their families and themselves [11,15,38].

In our study, 11 (out of 19) women had lost their jobs during or after treatment, mainly because their contract had not been renewed. In addition, we could observe the different situations that occurred among employed women with temporary and more precarious contracts compared with those who had indefinite contracts. The most protected situation was that of the six female civil servants of the public administration, which in Spain is a much more guaranteed and indefinite type of employment. Other studies have revealed the increased chances of job loss for people with temporary versus indefinite contracts [39]. The case of women employed in domestic service (five) was also particularly significant due to the lower incomes and the difficulties to subsist involved in periods of sick leave, because this regime offers less protection, it is usually occupied by women with less training and status, and it is highly feminized (98.31% female) [25]. The work of the self-employed people was associated with lower absenteeism after cancer and also with reduced protection compared to employees [37,40]. Greater difficulties were also identified in poorly educated women with low socio-economic status [21,41], who often access harsher physical jobs and have more unstable contracts [42] and less access to social rights and to information [43].

Although most of the women in our study indicated the desire and the need for jobs to be adapted to their circumstances in order to go back to work, these measures had not been taken, despite being one of the factors that the international literature has identified as promoting the return to work, especially in the case of physical and low-skill jobs [14,41]. In addition, the employers’ perceived mistrust, coupled with social self-stigma and perceived discrimination due to the disease, suggested to these women the dilemma of concealing their illness in job interviews, in line with the reports of other studies [15,44,45,46].

Eleven women in our study had to embark on the difficult path of finding a new job without having sufficient legal and administrative support for it. In other cases, the presence of sequelae for the work activity after the maximum period of disability sick leave ran out, led some women to request permanent disability, or early retirement if they were older than 55 and qualified for it (the two pre-retired women were civil servants).

Finally, our female survivors unanimously agreed that returning to work or starting a new job adapted to their physical and psychological conditions would require more assistance and support to deal with the complicated situations they face. Most of them had not received any advice about employment, or only very limited advice. They also pointed to a lack of coordination between the health system and the social protection systems, observing divergent assessment criteria concerning the extent of the sequelae and the capacities for work.

### 4.1. Strengths, Limitations, and Suggestions for Future Research

The main strength of this study is that it has allowed us for the first time to explore the problems and itineraries of going back to work from the perspective of women after treatment for breast cancer in Spain, where there is also a very small number of studies of the occupational impact of cancer in general. This provides valuable knowledge that must be taken into account by all actors of the health system, social security, employers, and actors involved in the process of rehabilitation and returning to work after breast cancer.

We are aware that this study has not allowed us to contemplate with sufficient breadth the different types of situations in terms of personal characteristics and variables, health, and employment, because our sample was small. However, we detected some of the problems encountered by women with cancer when going back to work in terms of their physical and psychological limitations, their difficulties and the social barriers to maintain or seek employment, and the lack of labor guidance and coordination between the services that attend to them, as well as disparities in the type of work, contracts, and socio-economic status. It is essential to continue research in this field, and it should be conducted in our setting, with samples incorporating survivors from different types of clinical situations, jobs, and socioeconomic status, as well as other actors and professionals of the health system, social security, social services, and the employers themselves.

### 4.2. Implications for Social Practice

From a practical point of view, returning to work must be addressed as a cross-sectoral issue involving different levels related to personal circumstances, the health system, the social security system, and the workplace. In addition, it should be considered as a process that occurs at different times, under different circumstances and itineraries, and that requires attention and implementation of strategies to adapt to the job so that it can be carried out successfully.

Based on the experiences of the women in our study, we suggest that there should be greater and better information and guidance for cancer patients about the impact of its physical and psychological consequences and about how to address going back to work or searching for a new job. Social workers put forth enormous time and energy to begin to understand the unique needs of people living with cancer [47], but now it is necessary to pay attention to return of surviving women as well as their difficulties and needs. Specific interdisciplinary programs and services should be offered early on to address the issues raised by female breast cancer survivors. Among them, we highlight the support and cooperation measures that facilitate the partial and gradual return to work or the search for new adapted jobs, the measures aimed at training and coordination among all the actors involved in this process, and the need to enhance legislative measures to comply with established rights.

## 5. Conclusions

The discourses of our survivors suggested that going back to work after cancer presents significant difficulties related to their individual physical and psychological circumstances, as well as to other factors of the institutional and work settings. This situation entailed implications for the survivors themselves, the various professionals, services, and administrations involved in their care, and the employers. From a political and welfare point of view, we consider it necessary to pay particular attention to the negative consequences of the evolution towards a labor market with increasingly temporary and precarious contracts, as well as to their impact on the protection and health and quality of life of the women affected by diseases such as cancer, who face double discrimination in the workplace, because they are women and they have cancer.

## Figures and Tables

**Table 1:** Categories resulting from the content analysis of the studies.

Primary Category (Theme)	Secondary Category (Sub-Theme)
Theme 1: Self-perception of cancer survivors	
Theme 2: Motivations to go back to work	
Theme 3: Going back to work: the pathways and difficulties	3.1. Going back to the “same job” 3.2. Searching for a new job 3.3. Early retirement

## Data Availability

The data presented in this study are available from the corresponding author on reasonable request.
